# Atlas-based spatiotemporal MRI phenotyping of 3D fungal spread in grapevine wood

**DOI:** 10.1016/j.plaphe.2026.100185

**Published:** 2026-03-30

**Authors:** Gargee Phukon, Maïda Cardoso, Christophe Goze-Bac, Loïc Le Cunff, Jean-Luc Verdeil, Cédric Moisy, Romain Fernandez

**Affiliations:** a UMR AGAP Institut, INRAE, F-34398, Montpellier, France; b CIRAD, UMR AGAP Institut, 34398, Montpellier, France; c BNIF University of Montpellier, Place Eugène Bataillon, Montpellier, France; d IFV, French Institute of Vine and Wine, IFV, INRAE, UMT Géno-Vigne, Institut Agro, 34398, Montpellier, France

**Keywords:** Grapevine trunk diseases (GTDs), Anatomical phenotyping, Time-lapse tracking, Magnetic resonance imaging (MRI), Probabilistic atlas

## Abstract

In perennial crops, inner wood degradation by pathogens often escapes detection until irreversible damage has occurred. Grapevine trunk diseases (GTDs) are a well-known example in viticulture that alter plants from within, years before foliar symptoms arise, making early assessment difficult. To overcome this limitation, we present a novel non-destructive 3D + t pipeline for high-resolution Magnetic Resonance Imaging (MRI) spatial quantification and monitoring of early internal host tissue degradation resulting from fungal pathogen colonization. The pipeline integrates spatiotemporal anatomical alignment and rigid registration; a generalized cylindrical-coordinate transformation; supervised segmentation of water-depleted regions; and population-level statistical analyses, including population mean images, probabilistic atlases, and 3D lesion descriptors. Applied to multiple *Vitis vinifera* cultivars inoculated with a fungal wood pathogen, our approach enables *in vivo* time-lapse comparisons between cultivars and treatments. The results reveal reproducible early degradation signals across individuals and cultivar-dependent differences in lesion progression. Overall, this methodological innovation provides a new paradigm for internal plant phenotyping, enabling non-invasive quantification of disease development and comparative spatiotemporal assessment of host responses in woody plants, with strong potential to advance early diagnosis and management of GTDs and internal diseases.

## 1 Introduction

Viticulture plays an important role in the cultural and economic heritage of the Mediterranean region and is a major agricultural sector worldwide. Recently, grapevine trunk diseases (GTDs) have become a significant challenge to the long-term sustainability and productivity of vineyards. Common GTDs, such as *Eutypa dieback*, Esca and Botryosphaeria progressively damage the internal woody tissues of grapevines, leading to declining vine health, reduced yields, and eventually vine death, causing significant economic losses for growers [1]. Detection and monitoring of GTDs involve tracking foliar symptoms like leaf discoloration or patterns. They are unreliable because their onset may occur within three to eight years after infection [2]. Hence, while visual scouting remains fundamental, it does not actually describe the internal health state of the specimen.

Traditional methods for controlling GTDs are often destructive, such as trunk removal, or rely on toxic fungicides still used in some countries but now banned in the European Union (EU). In this context, our objective is to develop non-destructive methods to characterize the early progression of GTDs in the internal tissues, quantify the disease development and differentiate early varietal responses to alternative treatment.

Because conventional approaches are destructive or unreliable, non-destructive imaging techniques have been brought forward to overcome this challenge. Multispectral and hyperspectral imaging have been proposed for automated detection of Esca-symptomatic vines [3], but these methods rely on temporary external factors that poorly reflect internal disease status. Mid-infrared (MIR) spectroscopy has also been explored for GTD detection [4], yet its diagnostic utility is constrained by shallow penetration depth and limited disease specificity.

All these approaches mainly focus on what can be observed externally or within a shallow layer of wood, whereas GTDs develop primarily in the deeper structures of the vine. Consequently, X-ray computed tomography (CT) and micro-computed tomography (microCT) [5,6] emerged as promising tools to study the internal structure of grapevine trunks. CT can reveal differences in radiodensity, and its micro-CT variant delivers exceptional spatial resolution, enabling precise identification of internal defects, including necrosis, decay, and black spots. CT provides a full 3D structural description of grapevine wood [7]; however, it does not capture the plant's physiology, including responses to pathogens and therefore is less effective at detecting weak signals associated with early stages of fungal infection, before structural degradation occurs and becomes detectable by CT. This limitation calls for complementary investigation techniques such as magnetic resonance imaging (MRI).

In plant biology, MRI has been used to observe both the static anatomy of internal tissues and dynamic processes like fluid flow. The primary advantage of MRI over CT is its ability to provide details about both the structure and the function of plant tissues. High-resolution 3D MRI images can be leveraged in plant imaging for measuring physiological parameters like water content, flow velocities, and tissue water status (mechanical and chemical water binding). MRI can be tuned to different modalities (e.g. T1-weighted, T2-weighted, diffusion weighted imaging) to highlight different aspects of plant tissues. For instance, T1-weighted images highlight watered tissue boundaries, while T2-weighted images are more sensitive to the water chemical and mechanical binding [7]. Recent advances in plant MRI have transformed the technique from largely qualitative visualization into a quantitative, non-invasive framework that enables integrated phenotyping of structural, hydraulic, and metabolic processes across scales [8]. Quantitative relaxation mapping has been used to resolve tissue-level water-status heterogeneity and dehydration effects in leaves [9]. Portable and low-field implementations are also progressing rapidly, with recent syntheses showing how in-situ MRI can measure plant structure, water content, flow, and stress/disease responses beyond laboratory constraints [10]. An inflow-based flow MRI method was applied to quantify slow xylem sap flow in tomato stems [11]. In parallel, contrast-specific developments, including lipid-sensitive MRI, extend plant MRI beyond water-based measurements toward non-destructive metabolic and storage compound phenotyping [12]. Numerous other applications have been developed in plant MRI research, including visualizing xylem embolism [13], monitoring root growth and development [14,15], quantifying water content and storage [16], and investigating stress physiology and drought responses [17].

Because MRI is non-invasive and non-destructive, it enables repeated monitoring of a specimen over time. It provides information on internal tissues without harming the plant, and its high sensitivity to water distribution and dynamics makes it a promising tool to detect physiological state changes and quantify infection responses. Based on these advantages, we hypothesize that MRI is suitable for non-destructive monitoring of internal tissues and quantitative characterization of grapevine cuttings from diverse varietal backgrounds. To test this hypothesis, we used MRI to monitor *in vivo* disease progression within grapevine trunks, generating and analyzing a four-dimensional dataset comprising 3D anatomical and functional information of specimens acquired at multiple time points. Ultimately, these efforts aim to enable early, non-destructive diagnostics and population-level characterization of trunk diseases using MRI. To develop a methodology for early GTD detection and quantitative characterization, we focused on visualizing and measuring pathogen-induced effects on water content and tissue status in xylem vessels, cambium, and wood rays surrounding inoculation sites. Thus, the objective of this present work is to establish a methodological framework and test its suitability for longitudinal *in vivo* monitoring of disease effects, while assessing differences in susceptibility among grapevine varieties to an Esca-associated pathogen.

We undertook a systematic evaluation of wounded specimens with and without pathogen exposure. To make these comparisons robust across specimens, we developed an automated image alignment pipeline that registers individual MR images into a common reference geometry. Given that grapevine trunks naturally vary in diameter, curvature, and anatomy, direct comparison of infection patterns across specimens is challenging. To address this, we propose a custom transformation framework to resample trunk cross-sections relative to the cambium contour in all the 3D volumes. We designed a generalized cylindrical transform to unwrap the curved cambium into a flat reference line, producing a standardized geometry across specimens. Such a normalized coordinate system is expected to enable radial (Δr) and tangential (Δθ) progression to be clearly separated, thereby highlighting whether pathogen spread occurs along the cambium or along the wood rays.

We hypothesize that this method can be automated and applied high-throughput to facilitate the quantification and comparison of complex circular infection patterns across populations. However, many studies that use advanced imaging and geometric analysis, including 3D + t geometry, are limited to a single specimen, or only a few individuals. This is often due to the complexity of experimental protocols, the challenges of large-scale data management, and the difficulty of comparing specimens that vary in size, shape, or geometry. As a result, most imaging-based studies lack statistical power and cannot generalize their conclusions to a population. Interestingly, population averaged images have been long used in atlas-building frameworks in biomedical imaging (such as the ICBM/MNI brain atlas [18]), and atlas frameworks became instrumental in computational anatomy. Recent imaging studies consistently rely on mean images to represent population-level signal patterns, including fetal [19] and neonatal MRI [20], and population based organ atlases [21]. However, the application of atlases to plant imaging is relatively new [22].

**Fig. 1:**
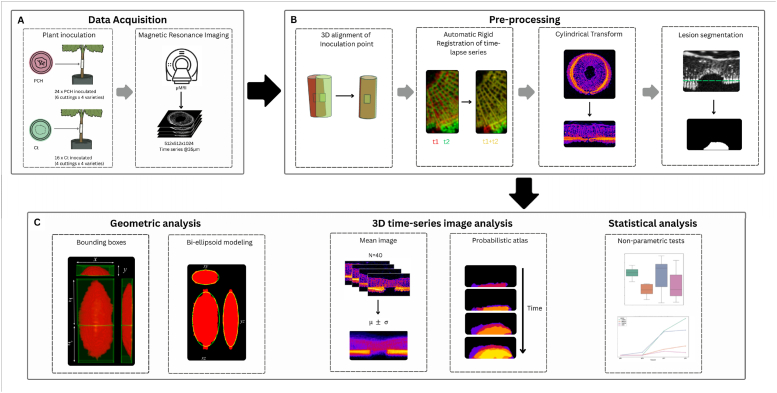
Schematic overview of the methodological pipeline. (A) Plant material was inoculated with the fungal pathogen or control and imaged at successive timepoints using μMRI. (B) The resulting 3D images were pre-processed through alignment of the inoculation point, automatic rigid registration of time-lapse datasets, cylindrical transformation of the trunk volume, and lesion segmentation. (C) Quantitative analysis was then performed using geometric lesion descriptors, including bounding box and bi-ellipsoid modeling. Group-level comparisons were then carried out using mean images, probabilistic atlases, and statistical comparisons to assess lesion development across conditions and varieties.

In this study, we present a methodological framework (see Fig. 1) combining 3D + t MRI, geometric normalization, and probabilistic atlas construction to enable population-level analysis of early grapevine trunk disease progression. As a proof of concept, we applied this pipeline to grapevine cuttings inoculated with *Phaeomoniella chlamydospora* and monitored lesion development over time across multiple cultivars. Using atlas-based representations and geometric lesion descriptors, we show that early internal tissue degradation can be quantified reproducibly and compared across individuals. Notably, these measurements reveal distinct early response patterns among cultivars, allowing the identification of response groups at early stages of infection.

Interestingly, the susceptibility ranking inferred from early MRI-derived lesion dynamics does not fully match the long-term classifications reported in the literature, raising new questions about the sequence linking early physiological responses and chronic trunk disease symptoms.

## 2 Materials and methods

### 2.1 Plant and fungal material

The experiment was conducted on 40 grapevine stem cuttings (ten repetitions for each of four cultivars). Each cutting was a one-year-old branch, approximately 15 cm in length, with two buds, bearing two to four leaves and displaying a well-developed root system. After pruning, cuttings were individually potted in plastic tubes designed to fit the micro-MRI chamber (40 mm diameter). The cuttings were maintained in a greenhouse at CIRAD, France under controlled conditions, including regulated temperature and humidity, supplementary lighting, automated irrigation, and preventive treatments against downy and powdery mildew.

#### 2.1.1 Vine cultivars

We selected four cultivars of *Vitis vinifera*, two red (Merlot, Tempranillo) and two white (Chardonnay, Ugni Blanc), representing contrasted responses to GTDs, based on external foliar symptom expression (see Table 1). These varieties are major European wine cultivars and differ in their susceptibility to Esca and its associated pathogen *Phaeomoniella chlamydospora* (Pch). By including cultivars considered relatively tolerant (Merlot, Chardonnay) or more susceptible (Tempranillo, Ugni Blanc), we designed the experiment to quantify various responses under infected and uninfected conditions.

**Table 1:** Grapevine cultivars selected for this study and their reported susceptibility to Esca.

Cultivar	BerryColor	Reported susceptibility to Esca	Reported susceptibility assessment with references	Notable features
Chardonnay	White	Low	<5% vines with foliar symptoms; almost no apoplexy over 8–14 years [23]	Rarely shows foliar symptoms; reported high resistance in long-term studies
Merlot	Red	Moderate	∼8% annual symptom incidence; ∼36% cumulative over 6 years; relatively low mortality [24]	Shows some foliar symptoms but at lower incidence; expected stronger compartmentalization of *Pch*
Tempranillo	Red	High	High frequency of foliar symptom expression; vine decline noted in Spanish vineyards [25]	Widely planted in Spain; repeatedly documented with high Esca incidence
Ugni Blanc	White	Very high	annually; up to 72% mortality over 6 years 30–44% vines symptomatic [26,27]	Among the most disease-prone varieties; consistently high incidence in French vineyards

While these descriptions cover long-term impact of infection on grapevines, in contrast, our experiment does not focus on chronic outcomes (or year-to-year symptom expression), but instead targets the early stages of fungal infection, caused by artificial inoculation of Pch. This raises a key question of whether the susceptibility gradient that becomes evident over years in the vineyard (especially with Chardonnay generally more tolerant and Ugni Blanc highly sensitive) translates into similar trends in the earliest physiological changes revealed by MRI.

#### 2.1.2 Fungal material

*Phaeomoniella chlamydospora* (Pch) is a vascular ascomycete fungus widespread in vineyards worldwide. It colonizes the xylem of grapevines [28], causing vascular damage that is characteristic of Petri disease, and constitutes a core component of the Esca complex in mature vines. Pch spreads through pruning wounds or graft unions, leading to vessel blockage, wood necrosis, and occasionally foliar symptoms or vine death. Unlike wood-decaying fungi, it does not degrade lignin or cellulose but impairs vascular function via toxin production and vessel occlusion. While host defenses such as phenolic accumulation and PR protein expression occur, they are often too delayed to prevent disease. Pch spreads both longitudinally through xylem vessels and radially/tangentially through tracheary tissues.

### 2.2 Wounding and inoculation

Inoculations were carried out following the protocol developed by the AGAP-DAAV team and described by Péros [29] and Moisy [30], with one modification: the 2 mm × 2 mm inoculation wound was made using a scalpel rather than a drill, to avoid wood burning and to improve consistency in wound size and depth. Branches were collected in December and stored at 4 °C. The day before inoculation, two-node cuttings were prepared (basal bud removed) and soaked in water overnight. For each cutting, a standardized 2 mm × 2 mm wound was created by removing the bark in the internodal region below the upper bud, approximately 3–4 cm from the top node. Among the ten wounded cuttings per cultivar (repetitions), six were inoculated with *Phaeomoniella chlamydospora* (Pch), and four received a control treatment. For inoculation, a 2 mm agar plug containing mycelium was cut from a fresh culture grown on PDA medium (10–15 d at 25 °C) and inserted into the wound with the mycelium facing the wood; the site was then sealed with plastic film. Control cuttings received sterile PDA agar plugs and were sealed in the same manner. This ensured that control vines experienced the same mechanical wounding and handling as inoculated vines, but without the presence of fungal pathogens. This experimental design allowed us to test the hypothesis that Pch-inoculated cuttings would display distinct internal symptom development, whereas control cuttings would primarily undergo normal wound healing, providing a clear contrast between pathogenic progression and tissue response to the wound.

### 2.3 Time-lapse magnetic resonance imaging

**Fig. 2:**
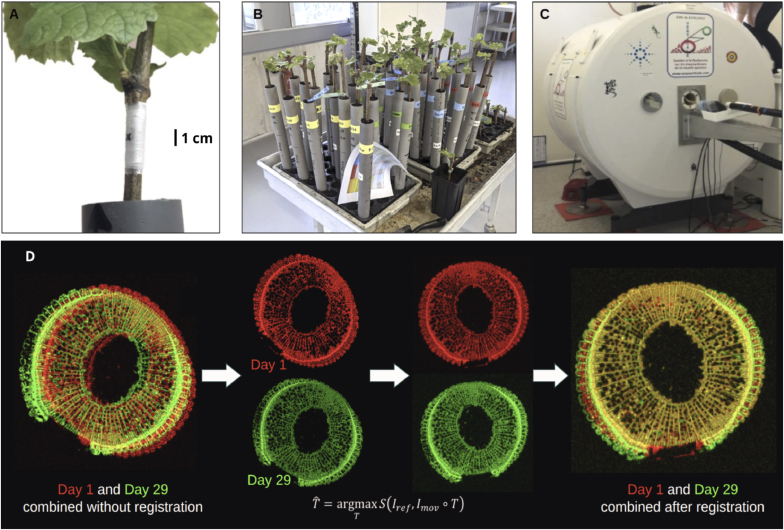
Experimental material, imaging setup, and effect of rigid registration on μMRI data. (A) Grapevine cutting showing the inoculation site after treatment (B) Total population stored in the greenhouse (C) MRI at BioNanoNMRI, Montpellier University (D) Representative transverse μMRI slices acquired at two timepoints (Day 1 and Day 29), shown before and after rigid registration. Overlaying images without registration highlights spatial mismatches, while rigid registration aligns internal structures, enabling voxel-wise comparison of tissue changes over time.

Once the cuttings were inoculated with Pch or control treatment, they were transferred to BioNano Imaging Foundry (University of Montpellier) for magnetic resonance imaging (MRI) (Fig. 2 (C)). Imaging was performed at four time points post-inoculation. The first scan (day 1 post-inoculation) served as a reference image, before visible symptoms development. The second scan (day 29) was expected to capture the early response phase, when localized functional changes associated to water availability in the tissues would begin to appear around the inoculation site. The third scan taken on day 77 post-inoculation was intended to monitor the mid-progression phase, a stage where more extensive tissue alterations were anticipated. The final scan on day 141 was chosen to represent the late stage of disease development, when the most pronounced internal alterations were expected. Collectively, these four time points yielded a time-lapse volumetric dataset (3D + t), allowing spatio-temporal analysis of both structural and functional changes within the same cuttings. The imaging was conducted between June and December 2024.

All MRI acquisitions were performed using a 9.4 T high-field MRI system with 35 μm isotropic resolution. This high-resolution scanner enables detailed anatomical visualization of wood integrity and tissue degradation. Three MRI modalities were employed: 3D Gradient Echo (GE3D), T1-weighted, and T2-weighted. In this study, we used only the GE3D data, which are high-resolution and isotropic, in order to foster an accurate geometrical description of the phenomenon.

T1-weighted and T2-weighted imaging approaches are optimized to differentiate tissues based on relative T1 and/or T2 relaxation times. In plants, these modalities reflect water relaxation, highlighting characteristics of water-rich compartments. In our setup, these sequences were acquired with very thick 3D slices, resulting in anisotropic voxel sizes (30 times larger along Z than along X/Y). This prevented reliable 3D characterization of the spatial progression of the pathogen and the tissue response over time. GE3D is an imaging sequence built upon the spin T1 and T2 relaxation processes and a flip angle. It enables an isotropic volumetric depiction of water-rich versus water-depleted tissues, offering a stable anatomical context for segmentation and analysis. The tradeoff is that the GE3D signal results from a combination of multiple weightings, making it ill-suited for pixel-wise quantitative MRI (e.g., a twofold signal increase cannot be interpreted as a twofold change in water density, chemical binding, or biological activity). Nevertheless, our analysis does not rely on pixel intensity proportionality, but rather on distinguishing living tissue from non-functional tissue, enabling quantitative analysis of the lesion-volume evolution over time. In this context, the capacity of GE3D to highlight living and water-containing tissues with an accurate three-dimensional anatomical-level description is instrumental.

GE3D acquisition parameters were TR/TE (Repetition time/Echo time) = 70/5 ms, flip angle = 30°, necho (number of echoes) = 1, FOV (Field Of View) = 36 × 18 × 18 mm^3^, and matrix (reconstructed image matrix size) = 1024 × 512 × 512, yielding a final voxel size of 35 × 35 × 35 μm^3^. The acquisition used a transverse volume orientation (vorient = trans (transverse); orient = sag (sagittal orientation)). The acquisition time per 3D volume was 1 h 16 min 28 s.

### 2.4 Pre-processing

Each of the 160 resulting 3D volumes (one per specimen per time point) contains over 1000 serial slices with a 512x512 definition. The images are 32-bit multi-slice TIFF files (1 GB per stack). Within these 3D volumes, the specimen appears with random orientations and inconsistent positions within the image coordinate system (x,y,z). To enable meaningful comparisons, a multi-step processing pipeline [31] was designed, consisting of normalization, registration, cylindrical transformation and segmentation.

#### 2.4.1 Normalization

To ensure consistent comparison of image intensities across specimens and time points, all MRI stacks were normalized using a water-filled capillary included in every scan as an internal reference. Voxel intensities were linearly rescaled relative to the median reference signal after subtracting background noise. This produced standardized image stacks where values are expressed in relative units with respect to the capillary, thereby reducing the influence of the device variability and enabling quantitative comparisons across time and treatments.

#### 2.4.2 Inoculation point alignment

Raw images differed in placement because samples were mounted manually, meaning the specimen would appear at slightly different positions and orientations across datasets and time series. The alignment step was intended to correct these inconsistencies by reorienting and repositioning each 3D image stack so that all specimens shared the same reference geometry, thereby bringing the inoculation window into a consistent location across stems relative to plant geometry. By treating the inoculation point as an invariant reference, we eliminated misleading sources of geometric variability across datasets and established a stable ground for analyzing patterns of growth and the effects of infection spread over populations. In practice, this alignment step estimates a 3D rigid transform (rotation + translation) that maps each volume into a common coordinate system before automatic registration, thereby reducing pose variability without modifying the shape of the specimen.

From each grapevine MRI scan, we manually extracted the real-world coordinates of three anatomical landmarks, the approximate midpoints of the trunk in the top and bottom slices, and the centre of the inoculation site. From these points, an orthonormal basis was constructed. The vector from the bottom to the top landmark defined the longitudinal axis of the trunk, ***v*_*z*_**. The inoculation point was then orthogonally projected onto this axis, allowing its position to be expressed in two parts: an axial component describing inoculation point height along the trunk, and a lateral component describing the depth in the trunk. The lateral offset vector defined the second axis, ***v*_*y*_**, while the third axis ***v*_*x,*_** was obtained by calculating the cross product, ensuring an orthogonal right-handed reference frame. Using this basis, a linear transformation was computed that reoriented the specimen into the desired axes and translated the inoculation point to a predefined target coordinate in voxel space (256,384,512).

#### 2.4.3 Rigid registration

**Fig. 3:**
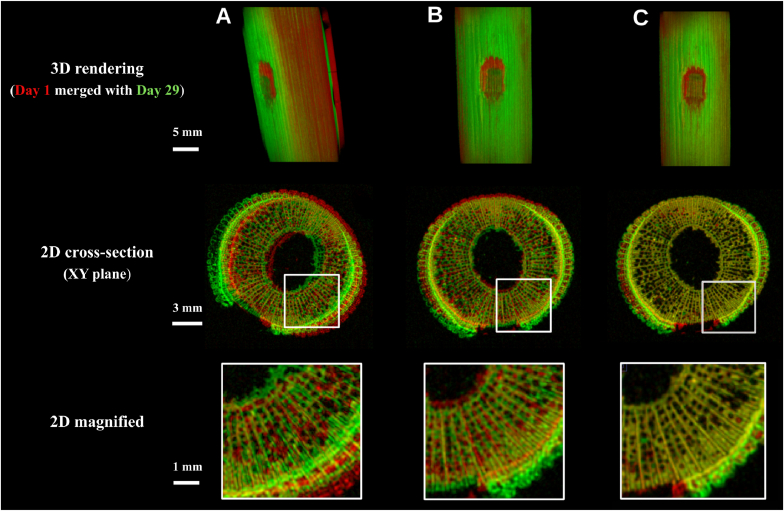
Results of geometric alignment and rigid registration on longitudinal μMRI data superposition. Superimposed μMRI data are shown (A) before processing, (B) after geometric alignment of the inoculation point to a common reference position, and (C) after automatic rigid registration. For each condition, the top row shows a 3D rendering of the trunk, the middle row shows a representative transverse cross-section (XY plane), and the bottom row shows a magnified view of the highlighted region. Improved spatial correspondence of internal structures and lesion boundaries is visible after rigid registration (pixels in yellow combine red (day1) and green (day 29), highlighting a good level of image correspondence). Scale bars: 5 mm (3D rendering), 3 mm (cross-sections), and 1 mm (magnified views).

We used automatic rigid registration with Block-Matching from the Fijiyama libraries [32] to align the different timepoints within each specimen time-course. While MRI signal intensities change during fungal progression, the wood tissue geometry remains stable over the time interval of the experiment. The biologically relevant variability occurs primarily within internal tissues, which can be meaningfully tracked once images are aligned in a common spatial framework. To that end, we used rigid transformation that includes only translation and rotation while preserving distances, angles, and anatomical proportions. As a result, the shape of the wood and its internal anatomical structures remain geometrically unchanged by the registration process (see Fig. 3), while the intensity of the corresponding tissues could change with time, during the ongoing progression of the pathogen, highlighting change in watering status, and allowing an accurate identification of pathogen progression. For regions that undergo substantial growth or bending-related deformation (e.g., shoots/branches or bark/cambium in whole plants), rigid alignment may be insufficient, and non-rigid registration would be more appropriate. In the present study, registration is applied to the mechanically stable internal woody compartment, so non-rigid registration was not required (see results section “Automatic rigid registration”)

Rigid transformations were estimated in Fijiyama by 3D block-matching (see Ref. [32] for an exhaustive description). A set of cubic blocks is sampled in the reference volume, and for each block, the corresponding location is searched in the moving volume within a bounded neighborhood. Candidate block correspondences are scored using the squared correlation coefficient (SCC) between the flattened 3D blocks, and the rigid transform is obtained from the set of matched block centers (least-squares rigid fit). This obtained transformation is then applied, and a new iteration begins to identify updated correspondences. This process is performed within a multi-resolution pyramid, starting with a coarse representation of the 3D images and progressively refining it at finer resolution. The registration parameters were set to: block half-size = 3 voxels, block spacing = 3 voxels, search neighborhood radius = 2 voxels, pyramid levels = [4,2,1]. The final transformation applied to each time point is the composition of inoculation-point alignment and block-matching registration.

Rigid registration (Fig. 2(D)) was performed in a daisy-chained manner to align all 3D volumes to Day 1, which was used as the reference scan. Day 29 was registered directly to Day 1. (t2→1), together with the inoculation alignment. Day 77 was first aligned to Day 29 (t3→2) and then mapped to Day 1 by composing t3→2 with t2→1, together with the inoculation alignment. Similarly, Day 141 was registered to Day 77 (t4→3) and brought into the Day 1 space via application of the composed transformation t4→3, t3→2, t2→1, and the inoculation alignment.

Following registration, each 3D image volume was stacked into a 4D hyperstack, where the Z-dimension represents the image depth (slices), and the t-dimension corresponds to the time points (Days 1, 29, 77, 141 post-inoculation). This final hyperstack enables synchronized temporal and spatial analysis across the different modalities and timepoints for each specimen. Finally, this 4D representation is used for voxel-wise aggregation across time and individuals (group-average images and probabilistic atlases), which requires the sub-voxel rigid alignment described above to ensure anatomical correspondence.

#### 2.4.4 Generalized cylindrical transform

**Fig. 4:**
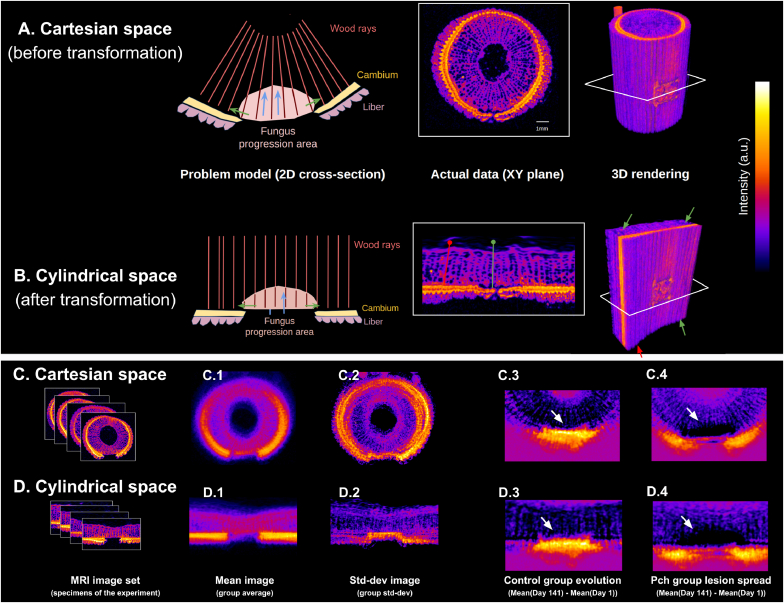
Comparison of Cartesian and cylindrical representations. (A) Illustration of the problem in Cartesian space, showing how fungal progression relative to anatomical structures (wood rays, cambium, liber) is distorted by specimen curvature, together with representative MRI data displayed as a transverse cross-section (XY plane) and a 3D rendering. (B) Corresponding representation after cylindrical transformation, in which the trunk geometry is flattened into a common spatial framework, enabling consistent localization of the wound and lesion region. (C) Analysis performed in Cartesian space, including mean images (C.1), the standard deviation map (C.2), the difference map in Control (C.3) and Pch (C.4). (D) Corresponding analysis in cylindrical space, showing improved spatial alignment and reduced geometric variability, resulting in clearer mean patterns (D.1), more interpretable variability maps (D.2), and the difference images (D.3 and D.4).

Woody perennials present considerable heterogeneity in size, shape, and tissue composition, making direct comparison across specimens challenging. However, grapevine cuttings exhibit distinctive elements of similarity: they have a cylindrical form and their cross-sections are almost a circle, that do not change within the experiment duration (the wood keeps its shape, even infected). To facilitate comparison across samples of different shapes and sizes, we unwrap these circular cross-sections into a flat representation using a cylindrical transform (2D polar transform along the Z axis). This transform has been widely used across disciplines whenever the underlying structure is circular or cylindrical, from unwrapping retinal [33] and endoscopic images [34], to recognizing contactless fingerprints [35]. By converting our (*x*,*y*,z) 3D images to cylindrical space (r,x’,z), we hypothesize that the complex circular progression patterns of the fungus around the cambium and into the xylem become easier to quantify (see Fig. 4 (A, B)). In this framework, radial direction (in depth, towards stem center) and tangential directions (along the stem axis or along the cross-section contour) are defined in a simplified manner, providing a basis for analyzing potential effects of infection spread along the denser wood rays or across it.

However, the cross-sections of grapevine cuttings are not perfect circles. These non-uniformities introduce inconsistencies that a standard polar transform cannot handle. To address this limitation, we developed a ‘generalized’ cylindrical transform algorithm that aims to adapt to irregular geometry and produce more consistent and comparable maps of fungal progression. The sequence of 3D images was transformed from the Cartesian space to cylindrical space by resampling along a reference cambium contour manually delineated on the central slice of the Day 1 volume. This reference contour was then replicated along z to define the cylindrical space coordinates consistently across slices. Formally, the cylindrical representation is a 3D volume indexed by (r, x’, z), where z is a coordinate along the trunk axis (slice index), x’ is the curvilinear coordinate along the contour, and r is the inward radial distance from the cambium toward the stem center. On the slices, x’ was expressed in voxels along the contour (arc-length sampling). It was discretized by sampling the cambium contour at approximately 1-voxel arc-length steps. The x-dimension range length was set to half of the cutting perimeter, which is, by a large margin, enough to cover the whole area of interest. The x’ window was centered on the inoculation site, so that x’ = 0 corresponds to the angular position of the inoculation point. For each sampled contour point, the inward direction was defined as the unit vector pointing from the contour point to the slice-wise trunk axis location, and r-sampling was performed along this ray with 1-voxel steps until reaching a fixed maximum depth (3.5 mm, which corresponds to the mean depth of the pith contour). Each resampling plane was defined with its origin on the contour and oriented towards the trunk's central axis, such that the successive planes intersect at this axis. Resampling was performed using trilinear interpolation on the original isotropic grid. The same geometric mapping was applied to all registered time points of a given specimen to ensure that each (r, x’, z) index corresponds to the same anatomical location over time.

As the trunk surface is not perfectly circular, the tangential direction was not strictly orthogonal to the radial plane. As x’ followed the contour arc-length rather than an angular coordinate, local metric distortions were expected, adding to the fact that pixels closer to the center corresponded to smaller circumferences and therefore appeared stretched in cylindrical space, forming trapezoidal regions. Consequently, the target pixels in cylindrical space did not correspond to uniform square pixels. To compensate for this distortion, we computed a “shrinkage map” that composed the shrinkage factor relative to the distance to the cambium (proportional, with value 1 at the cambium, and 0 at the stem axis) and the shrinkage factor relative to the angle between the cambium and the resampling plane (sine of the angle). This map stored, for each pixel, the appropriate surface-area factor required to account for its true physical size. This correction was essential for ensuring that subsequent surface or volume computations accurately reflected the underlying tissue geometry. We applied this map as a per-pixel weight when computing area-dependent quantities like lesion surface and volume, to recover physical units.

The resulting output was therefore a rectified “unwrapped” map (Fig. 4(B)), where the cambium corresponds to r = 0 and forms a straight line over x’ and a straight line over z. This flattening step was also intended to increase the effective comparability of samples, preserving more of the true signal needed to distinguish subtle differences in fungal behavior between varieties. In Cartesian space, size differences introduced high voxel-wise variance in the population of images when computing mean image, thereby masking biological significance. In contrast, the cylindrical transformation is designed to normalize the geometry, producing more consistent data (area of interest, including wound and lesion progression, share a same geometrical support) in which variability across specimens becomes more informative about the response to the fungal pathogen, and less about the different shapes of the cuttings.

The transformation was performed by sampling points along the cambium of the grapevine trunk with unit spacing between two successive resampling planes. Each resampling plane was defined with its origin on the contour and oriented towards the trunk's central axis, such that the successive planes intersected at the axis. As the trunk surface is not perfectly circular, the tangential direction is not strictly orthogonal to the radial plane. Consequently, the target pixels in polar space do not correspond to uniform square pixels. Pixels near the cambium are mapped with minimal distortion, whereas pixels closer to the center correspond to smaller circumferences and therefore appear stretched in polar space, forming trapezoidal regions. To compensate for this distortion, we compute a “shrinkage map” (using a sinθ factor) which stores, for each pixel, the appropriate surface-area factor required to recover its true physical size. This correction is essential for ensuring that subsequent surface or density computations accurately reflect the underlying tissue geometry.

#### 2.4.5 Segmentation

**Fig. 5:**
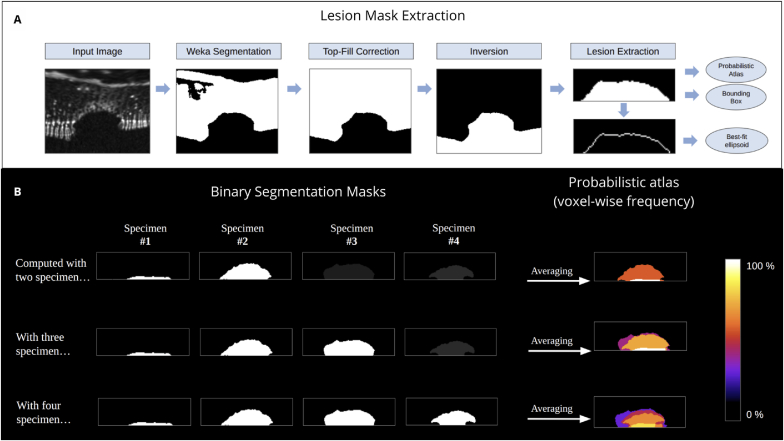
Lesion mask extraction and principle of probabilistic atlas construction. (A) Overview of the lesion segmentation and extraction pipeline, starting from the input MRI slice and proceeding through supervised Weka-based segmentation, top-fill correction, inversion, and final lesion mask extraction. The resulting binary masks were subsequently used to derive geometric descriptors (bounding box and best-fit ellipsoid) and to construct probabilistic atlases. (B) Generation of probabilistic atlases from binary lesion masks. Individual segmentation masks from increasing numbers of specimens were combined by voxel-wise averaging to estimate the frequency of lesion occurrence at each voxel.

The fungal colonization front cannot be directly observed in the MRI images. However, early fungal activity causes local tissue degradation that results in a loss of MRI signal, reflecting the loss of water within the wood and a loss of conductivity. This signal loss defines a lesion developing around the inoculation point. We therefore target to segment this lesion, which is a dark, low-intensity area replacing the bright signal associated with healthy, water-rich wood (see Fig. 5 (A)).

As a first step, a machine learning based segmentation model was trained to distinguish watered regions (bright) from non-watered (dark) regions, including dry tissue and background. To train the model, a representative subset of the dataset was assembled. Because lesion development is localized around the inoculation site, three axial slices per specimen were retained for training at each treatment and time point. For each treatment and time point, the selected slices from all specimens were gathered and served as training data for a Fast Random Forest classifier (200 trees) using the Trainable Weka Segmentation [36] libraries in ImageJ. The classification relied on a limited set of multi-scale intensity and edge-based features (Gaussian blur, difference of Gaussians, Sobel gradient, and Hessian filters), selected to reflect the strong contrast between water-rich and water-depleted regions. Model training and evaluation were performed using the automated cross-validation procedures provided by the Weka framework, yielding a very low Out-of-bag error. This error measurement was not used for evaluation, as the out-of-bag only characterizes the performance on the selected annotation points, which tends to not be representative of the data diversity in this GUI-assisted machine learning approach. We instead verified the output results: the trained model was applied to the complete dataset to generate binary lesion masks for all specimens across varieties, treatments, and time points, and the obtained segmentation was verified and corrected when needed, during a systematic screening of the slices.

Due to the pixel-based classification approach of the Trainable Weka Segmentation, low-intensity tissue pixels were often misclassified as non-tissue. To overcome this limitation and obtain the desired masks, we applied a post-processing step based on slice-wise top-fill correction. This approach is straightforward in our setup because, after the cylindrical transform, the upper rows of each slice consistently correspond to tissue, whereas the lower rows represent background. The correction involved identifying the first row with complete (or sufficiently high) tissue coverage and flood-filling all rows above it, ensuring a consistent tissue region. This resulting segmentation provided the basis for subsequent analyses of lesion geometry and progression.

### 2.5 Quantitative image analysis

The previous preprocessing steps were intended to provide a common geometric framework and a consistent localization of the disappearing region, the lesion, across all specimens. Building on this standardized representation, we developed quantitative tools to analyze how the phenomenon varies within and between populations of specimens, particularly between Control and Pch groups and across the different grapevine varieties. To achieve this, we developed aggregation strategies based on mean images and probabilistic atlases, which summarized group-level spatial patterns of lesion growth. We then defined a feature space to describe the 3D + t objects to characterize each specimen with a small number of interpretable parameters. These features formed the basis for the statistical comparisons performed later in the study.

#### 2.5.1 Population-based atlas construction

As stated in the introduction, atlas-based approaches are widely used in medical imaging and developmental biology to compare, combine, and quantify complex anatomical structures in 2D, 3D, or 3D + t. These strategies provide population-level descriptions that capture both common patterns and individual variability. In this work, after establishing a common geometric reference frame, we adopted an atlas-based methodology to achieve population-level anatomical phenotyping of the growing lesion.

##### 2.5.1.1 Mean image

Mean images (group-average images) were computed by voxel-wise averaging of the aligned and transformed MRI volumes to obtain a representative signal distribution for each group. Let $I_{k}\left(v\right)$ denote the signal intensity of the specimen k at voxel v, with $k=1,\ldots ,N_{specimens}$ ($I:R^{3}\to R$ associating an intensity value to each voxel v). Then the mean image μ(v) can be defined as:

(1) $$\mu \left(v\right)=\frac{1}{N}\sum_{k=1}^{N_{specimens}}\left(I_{k}\left(v\right)\right)$$

This averaged image captures the first-order trend of the population, reducing specimen-specific noise while preserving the recurrent patterns. Standard deviation maps were also computed to quantify variability around the mean and complement the mean image by highlighting regions where specimens differ.

##### 2.5.1.2 Probabilistic atlas

We constructed a probabilistic atlas that estimates, for each voxel, the likelihood of observing a given tissue state across a group of specimens. The atlas is generated by computing the voxel-wise frequency of lesion occurrence normalized by the number of specimens. We visually assessed the resulting atlas by examining XY and XZ cross-sections and the resulting mean image by analysing the distribution of the MRI intensity values and spatial patterns across individuals.

#### 2.5.2 3D + t specimen-wise geometric feature extraction

**Fig. 6:**
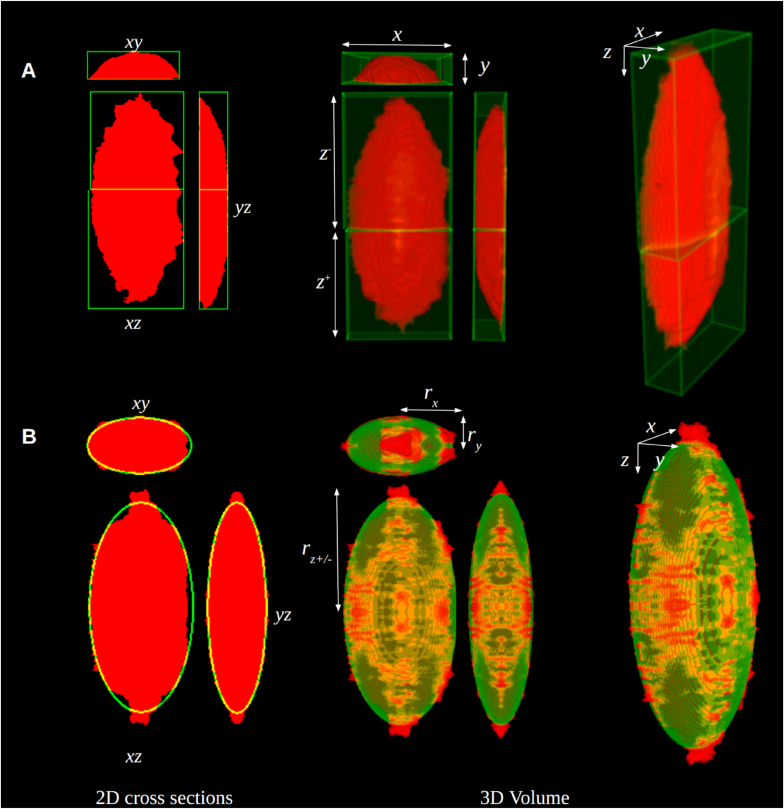
3D visualization of the segmented lesion. (A) Lesion geometry represented using axis-aligned bounding boxes. Orthogonal 2D cross-sections (xy, xz, yz) and a 3D rendering illustrate how the bounding box captures the overall spatial extent of the lesion along the longitudinal (z) and transverse (x, y) axes. (B) Corresponding representation using best-fit ellipsoid modeling. Ellipsoids were fitted to the binary lesion masks to provide a compact and interpretable description of lesion shape and anisotropy.

While atlas-based representations captured population-level trends, they do not directly quantify the geometric properties of individual specimens to compute statistical comparisons based on these values. To enable geometric comparisons between groups, we extracted a specimen-wise set of interpretable 3D + t geometric features from the segmented lesions. These features helped quantify how the affected wood volume/surface evolves spatially, as the deteriorated region is expected to enlarge over time. From each binary mask, we computed its bounding box (see Fig. 6 (A)) to capture the overall lesion extent and we fit an equivalent ellipsoid to the mask to characterize its 3D shape and anisotropy. These 3D + t parameters were then used for characterizing differences between populations, allowing downstream comparisons of lesion progression between Control and Pch treatments and across varieties, at both the inter-variety and intra-variety levels.

##### 2.5.2.1 Specimen lesion bounding box

We defined axis-aligned 3D bounding boxes around the lesion mask volumes to characterize them with a few parameters. The separation of the lesion volume into two halves was necessary because the progression of water disappearance tends to spread unevenly across the Z directions, the impact being often bigger on the upper tissues. To that end, these masks were divided at the centralmost Z slice (z = *cz*) to separate the volume into two complementary halves. The Z^−^ region, comprises slices below the inoculation plane (*z < cz*), and the Z^+^ region comprises the slices above it *(z ≥ cz).* This convention follows the standard geometric notation where the negative and positive indices represent opposite sides of a reference plane.

For each half-volume, an axis-aligned bounding box was computed by scanning the non-zero voxels of the segmented lesion binary mask to determine the minimum and maximum coordinates along *x*, *y* and *z*. Specifically, *x*_min_ and *x*_max_ correspond to the leftmost and rightmost lesion voxels, *y*_max_ to the highest extents in the vertical direction (cross-section-wise), and *z*_min_ and *z*_max_ to the first and last slices containing lesion voxels.

##### 2.5.2.2 Specimen lesion equivalent ellipsoid

In addition to the bounding-boxes, we aim to describe the lesion with geometrical features that better reflect its actual shape. For this purpose, the segmented lesion volumes were modeled by fitting 3D ellipsoids defined by the parameters *(x*_*0*_*,y*_*0*_*,z*_*0*_*,r*_*x*_*,r*_*y*_*,r*_*z*_), because the lesion volumes tend to exhibit such geometry. As the inoculation point lies on the outer surface of the wood, the lesion progresses only towards the centre and not outwards, resulting in an asymmetrical geometry, forcing us to estimate “half-ellipsoids”. Moreover, the lesion may develop differently above and below the inoculation point along the longitudinal axis of the cutting, which motivated the use of a bi-ellipsoid model (an ellipsoid to represent the upper half of the lesion and an ellipsoid to represent the lower half).

To implement this, each half (Z^+^ and Z^−^) of the lesion mask was mirrored along the *y* and *z* axes, producing double-symmetric volumes that approximated ellipsoidal shapes suitable for further geometric fitting. To obtain the desired equivalent ellipsoid representation of each double-symmetric lesion volume, we first extracted the contours of the lesion mask volume and retrieved the corresponding surface voxels, that is the point set ${\left(\{x_{i},y_{i},z_{i}\right)\}}_{i=1}^{n}$ defined as the coordinates of lesion voxels that neighboured at least one non-lesion voxel in the 6-connected directions. As the lesion is mirrored, the volume exhibits symmetry across both the *y* and *z* axes, cancelling out the cross-product moments *(xy,yz,xz)*, resulting in a diagonal inertia matrix with no rotation terms. We determine the ellipsoid parameters solving a least-squares problem using singular value decomposition (SVD) that identifies the set of parameters that minimizes the overall fitting error, yielding the ellipsoid centre *(x*_*0*_*,y*_*0*_*,z*_*0*_*)* and three radii *(r*_*x*_*,r*_*y*_*,r*_*z*_*).* By construction*, y*_*0*_ and *z*_*0*_ are the same for all ellipsoids, due to the double mirroring around the inoculation point. The radii described the spatial extent of the lesion along each axis, providing a quantitative measure of how far the infection had spread in different anatomical directions. As a final step, we evaluated the fit quality using residual metrics to ensure the fitted ellipsoids provided an adequate geometric approximation.

## 3 Results

The results are presented in two parts. First, we report the methodological results, evaluating whether each part of the pipeline performs as expected. Second, we apply the validated pipeline to characterize fungal progression within cuttings and quantify the differences between treatment groups and cultivars.

### 3.1 Methodological results

This section presents the methodological outputs of the pipeline, including alignment to the inoculation point using geometric references, rigid-body registration, cylindrical transformation, segmentation of the disappearing signal, and the construction of population-level models such as mean images and probabilistic atlases. We also report the geometric descriptors extracted from each lesion, namely bounding boxes and equivalent ellipsoids, which are later used for quantitative comparisons between groups.

#### 3.1.1 Inoculation point alignment

Manual alignment was based on three landmarks indicating the inoculation point and stem axis. We evaluated the effectiveness of this alignment by calculating the distance between corresponding anatomical points in scans of the same specimen over time. To that end, we used the Evaluate Mismatch functions in the ImageJ Fijiyama plugin [32]. The mean displacement between matched points after this first registration step was approximately 3 pixels in X, 7 px in Y and 0.2 px in Z, indicating that most residual variability occurred in the section plane rather than along the stem axis. In real-world units, these offsets correspond to roughly 0.10 mm, 0.25 mm, and 0.007 mm, respectively. Compared to the inoculation wound size (2 mm × 2 mm), these offsets are really small. The largest mismatch (Y) represents 12% of the wound size while the axial offset is negligible (<1%). When compared to the average cutting diameter (15 mm), the same mismatches represent <2% of the stems diameter, showing that the global orientation is preserved at the specimen scale. The overall 3D displacement averaged ∼0.27 mm which is equivalent to about 8 voxels given our isotropic voxel size of 0.035 mm. This indicates the inoculation-based alignment standardized orientation but left residual offsets that limit precise voxel-wise comparison.

#### 3.1.2 Automatic rigid registration

After applying rigid registration, mean displacement was reduced to 0.30 px in X and Y and 0 px in Z, corresponding to a maximum of ∼0.01 mm in each direction, and the total 3D distance dropped to ∼0.02 mm, or approximately half a voxel. Rigid registration improved alignment accuracy from ∼8 voxels to <1 voxel, achieving sub-voxel correspondence of internal anatomical structures. These results suggest that this hierarchical registration strategy combining alignment and automatic block-matching produces a sufficiently consistent framework to support voxel-wise anatomical comparison.

### 3.2 Generalized cylindrical transformation

By construction, the cylindrical transformation performs the “flattening” of the cambium tissue, from a cylinder to a flat plane. However, one limitation lies in the fact that the cross-sectional profile of the resampling cylinder is extracted manually from the central slice at the wound center level. As the object contour is not perfectly invariant along the Z axis, at some distance of the wound the contour shape is slightly changing. As a consequence, after the transformation the cambium appears less flat at a distance from the wound (Fig. 4 (B), red arrow). However, this concern appeared to be unimportant as visual investigations showed that the distorsion is very limited (Fig. 4 (B), green arrows) in the area under study (direct surroundings of the wound).

We investigated the quality of separation between radial and tangential axes by measuring the mean deviation of the orientation of wood rays relative to the expected orientation (vertical), on a random set of 10 images from the four varieties (28 rays measured for each image). After transformation, we measure an almost vertical mean orientation (89.1°), and a 3.07° mean deviation from the vertical axis (see Supplementary materials S.1). These results indicate an effective and unbiased reorientation of rays, while the separation of tangential and radial directions is imperfect, suggesting that the generalized cylindrical model covers most of the cutting geometry, but not totally.

Conceptually, the transformation worked as intended. However, because the fungus remained highly localized, the geometric changes introduced by the transform were limited. Still, this approach could become more informative in longer monitoring setups, or with other pathogens or hosts that show different spread behaviors.

### 3.3 Segmented masks of lesion

The segmentation step produced binary masks of the disappearing regions across all specimens, treatments and time points. The classifier separated bright (water) tissues from dark (non-water) tissues while the top-fill correction recovered tissue pixels that were initially misclassified as background, restoring a 3D tissue region across slices that appeared to be continuous and anatomically consistent in our visual verifications (see in Fig. 5 (A)). The resulting masks were then inverted and axially cropped around the inoculation point to isolate the region of interest along the stem. These final lesion masks delineated the zones of signal loss, which then served as inputs for atlas construction and bounding box extraction. The boundary pixels of these lesion masks were then extracted as surface points for equivalent ellipsoid fitting. The quality of the masks was verified by merging each segmentation with the original image and visualizing the overlap. In infrequent cases (3 images over 160), segmentation exhibited large discrepancy with the actual contour of the lesion area, and the slice-wise correction was done manually.

### 3.4 Atlas construction

#### 3.4.1 Mean images

After spatial alignment, registration, and cylindrical transformation, the images of all specimens in each group were averaged voxel-wise. In the Cartesian space, the average image (Fig. 4 (C.1)) appears blurred because the cross-section of the specimen differs in shape and size. This variability increases the pixel-wise standard deviation and smooths the mean signal, hampering the detection of time-lapse spatial trends in the population. After cylindrical transformation, the structures of interest align along a straight axis, resulting in much sharper mean images, with unscattered high intensity area (cambium) and a lower mean variance (Fig. 4 (D.1)). The area of high variance we are interested in studying, that appears around the infection zone, is well demonstrated in the cylindrical space (Fig. 4 (D.2)) highlighting the phenomenon under study, and producing cleaner, more interpretable population-level representations.

Finally, the difference images computed between time points (day 141 and day 1) reveal similar signal patterns in both Cartesian (Fig. 4 (C.3-4)) and cylindrical (Fig. 4 (D.3-4)). Although a stronger contrast enhancement was initially expected in cylindrical space, this was not observed. On the positive side, this result indicates that the cylindrical transformation does not introduce major distortions or artifacts in the signal, and preserves the underlying image content while providing a normalized spatial framework suitable for population-level comparison. Difference images are therefore used here primarily as a qualitative validation of the transformation rather than as an independent source of contrast between using cartesian space and cylindrical space.

#### 3.4.2 Probabilistic atlas

The probabilistic atlases were generated by combining the binary lesion masks and computing, at each voxel, how often the disappearing region (in white) occurred across specimens. The voxel-wise frequency produces a probability map that captures the spatial trends of the lesion geometry within the population. The probability values range from dark purple (0%) to bright yellow (100%), indicating how frequently the lesion appears at each location relative to the inoculation point across the population.

Fig. 5 (B) illustrates this process, showing how individual lesion masks were aggregated into a population-level atlas that highlights regions where lesions overlap consistently across the population (high probability) and regions where specimens diverge (lower probability). Computed with two masks, the atlas shows only three possibilities: 0% (no lesion), 50% (one of the two), or 100% (both). With three masks, the map gains intermediate values, revealing partial overlap (33% or 66%) and full overlap (100%). As more specimens are added, the atlas highlights the population patterns including both common regions (the overlap area) and variable regions of the lesion population. These atlases make it possible to identify group-level trends and to compare how consistently different groups develop the lesion (detailed in the following sections). We applied this to multiple groups to assess the differences: first, Pch group versus Control group (all varieties included), and then one group per variety, including only Pch infected cuttings, see section “Application to monitor and quantify differences between treatments and cultivars”.

### 3.5 Geometric features

#### 3.5.1 Specimen lesion bounding box

The bounding-box measurements provide a quantitative description of the spatial extent of the lesion in 3D. For each specimen, the box dimensions (height “z”, width “x” and depth “y”) reflect how far the affected region spreads along the different anatomical planes of the cutting. Concisely, larger bounding boxes indicate broader deterioration of internal tissues, while smaller boxes indicate limited/localized progression.

In Fig. 6 (A) the volume in red corresponds to the binary lesion mask, representing all voxels that lost MRI signal due to the fungal colonization. This volume is enclosed by an axis aligned bounding box (in green) which captures the outermost spatial extent of the lesion. Rather than describing lesion volume directly, the bounding box quantifies how far the lesion has spread in each anatomical direction, providing a geometric summary of its lateral, radial, and axial expansion. The x-dimension reflects tangential spread along the cambial region. The y-dimension captures radial penetration toward the trunk centre. The separation of the longitudinal axis into positive and negative z-directions allows independent measurement of apical and basal advancement. Together, these dimensions characterize the 3D extent of the lesion and reveal directional patterns in how the disease progresses through the cutting. However, the bounding-box captures the extremities, making it sensitive to abnormalities that do not correctly represent the actual lesion shape.

#### 3.5.2 Specimen lesion equivalent ellipsoid

To counter the limitation of bounding boxes, geometric ellipsoids were fitted to the contour of the lesion (Fig. 6 (B)). The average point-wise distance between the actual contour points and the estimated ellipsoids over all specimens is 2.62 ± 0.91 pixels (compared with the average ellipsoid radii extending between 25 and 100 pixels). This indicates that the fitted ellipsoids closely followed the true lesion contours. Only a small number of samples (3) exhibited high values, reflecting instances where the lesion geometry was more irregular and not well represented by a 3D ellipsoid. Overall, these results confirm that the ellipsoid model provides an adequate geometric representation of the lesions, useful to model their anisotropy with respect to the three anatomical axes.

To ensure that the ellipsoid approximation remained consistent with the lesion geometry, ratios between the ellipsoid radii and the corresponding bounding-box dimensions were also calculated. These ratios provided a simple check that the ellipsoid and the bounding box are not diverging excessively. In our data, the values remained close to 1 across axes, indicating that the fitted ellipsoids captured the lesion shape well and did not underestimate or overinflate its dimensions. This verification step confirms that the fitted ellipsoids are consistent with bounding boxes. However, ellipsoids appeared as a slightly better fit for representing the actual lesion geometry, and were considered more reliable and suitable for subsequent statistical analyses.

### 3.6 Application to monitor and quantify differences between treatments and cultivars

This section entails the comparison between populations of cuttings, by leveraging the methodology.

#### 3.6.1 Pch vs control

##### 3.6.1.1 Visual investigation of probabilistic atlases

**Fig. 7:**
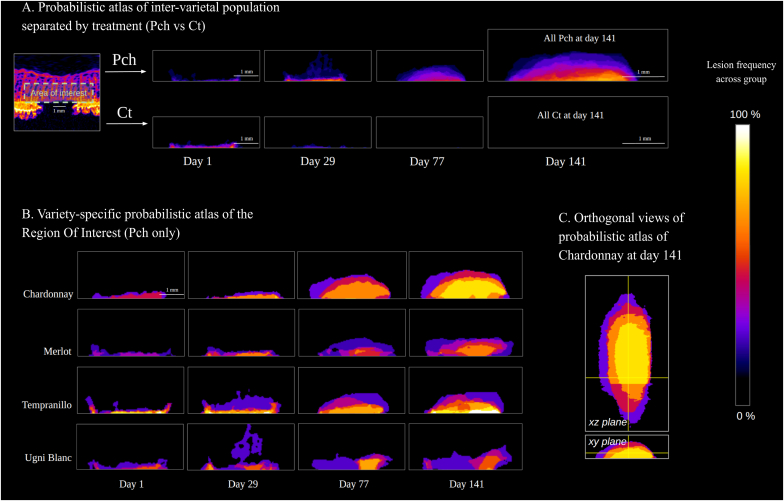
Probabilistic atlases of lesion occurrence across treatments and varieties. Color intensity indicates the voxel-wise frequency of lesion occurrence within each group, highlighting progressive lesion expansion in *Phaeomoniella chlamydospora*-infected samples compared with minimal signal in controls. (A) Probabilistic atlases of the region of interest aggregated across all grapevine varieties, shown separately for Pch-infected and Control specimens at successive time points (Days 1, 29, 77, and 141). (B) Variety-specific probabilistic atlases computed from Pch-infected specimens only, illustrating distinct temporal patterns of lesion development among Chardonnay, Merlot, Tempranillo, and Ugni Blanc. (C) Orthogonal views (xy and xz planes) of the probabilistic atlas for Chardonnay at Day 141, illustrating the three-dimensional spatial distribution and extent of lesion occurrence. Scale bars: 1 mm.

Fig. 7 demonstrates atlases where the colours encode how often a voxel is classified as a lesion across the population, from 0% (dark purple/black) to 100% (bright yellow/white). Only voxels above the cambium central line are considered for the analysis, as voxels below this line correspond to outer tissues (liber, bark), which are not under study.

The Pch group (infected) shows (Fig. 7 (A)) progressive build-up of high probability necrosis above the threshold (inoculation point) over time, entering the region of interest (inner wood) at the third timestep (day 77) and developing inwards at day 141, with shades of probability indicating a wide distribution of infection depth across the total population of Pch cuttings. Meanwhile, the Control group remains mostly low probability and fades with time, consistent with wound healing and the absence of internal degradation, ultimately exhibiting no deteriorated inner wood at all at the end of experience (day 141).

##### 3.6.1.2 Lesion quantification using ellipsoids

The ellipsoid lesion volumes were computed for each timepoint to quantify how lesion size evolved over the course of the experiment. Each lesion was modeled by fitting separate ellipsoids to the Z^+^ and Z^−^ regions along the stem axis, and their volumes were averaged to produce a single equivalent ellipsoid volume per specimen. This yielded one robust lesion-size estimate per specimen per timepoint, allowing the temporal progression of lesion growth to be compared across cultivars.

Fig. 7 (A) shows that Pch lesions, on average, develop slowly during early infection and then expand rapidly with substantial tissue damage accumulating between day 29 and day 77. In contrast, the absence of a similar volume in Control specimens (segmented volume = 0) further supports the fact that the phenomenon observed was a result of the pathogen action and not linked to the wounding, cultivation setup or imaging protocol.

#### 3.6.2 Cultivar differences

##### 3.6.2.1 Visual investigation of probabilistic atlases

We first examined the probabilistic atlas to extract the intra-cultivar variability (Fig. 7 (B)):

• Chardonnay and Tempranillo appeared to be the most consistent varieties, as reflected by probabilistic atlases showing an unscattered area, and the highest ratio between the surface of high probability (common pattern) and the surface of low probability (anomalies).
• On the other hand, Merlot and Ugni blanc exhibit a wide extent of the lesion for a few specimens, while the high probability is limited (in Ugni), or completely absent (in Merlot, the area with maximum probability is at 50%).

We then examined the intra-cultivar trends:

• Chardonnay shows the widest and most consistent high probability region around the inoculation zone (bright yellow, 80% probability, meaning almost all specimens show necrosis in these areas). The orange and red zones extend further towards the pith than in other varieties, indicating a consistently deeper lesion across the cultivar group. In 80 % of the Chardonnay specimen, the lesions reached at least 60% of the biggest lesion depth observed across the population at day 141. Along the x axis, the lesion reached additional tissue from the inoculation area, however this is the case for less than 50 % of the specimen.
• In Tempranillo, all the specimens have developed lesions in the direct proximity of the inoculation area. Along the y axis (depth) the specimen shows a gradient of reactions, the atlas showing a “stairs” pattern with a progressively decreasing lesion probability. Along the x axis (cambium), the lesion reaches no additional tissue outside the inoculation x-axis bounds, making the wood ray appear as an “invisible wall” in tempranillo.
• Merlot and Ugni Blanc hardly exhibit any trends, as their probabilistic atlases show scattered patterns. The lesions show minimal progress along the y axis, highlighting no common zone reached by all specimens, as a result of variation, with some showing a wide lesion development, and other showing no development at all. Ugni Blanc shows the smallest high probability area among all varieties. Most of the atlas is dominated by purple and magenta, corresponding to low probabilities (10-20%). This colour distribution suggests that most Ugni Blanc specimens experience limited tissue degradation and the lesion rarely expands from the inoculation point. However, both in Merlot and Ugni, some specimens show that once the fungus crosses the cambium surface, the lesion can spread outside the inoculation x-axis bounds, eventually through the wood rays. This effect is particularly visible in Ugni, with three specimens showing a localized infected area, while in Merlot a single specimen is concerned by this aspect.

##### 3.6.2.2 Lesion quantification using ellipsoids and bounding-boxes

**Fig. 8:**
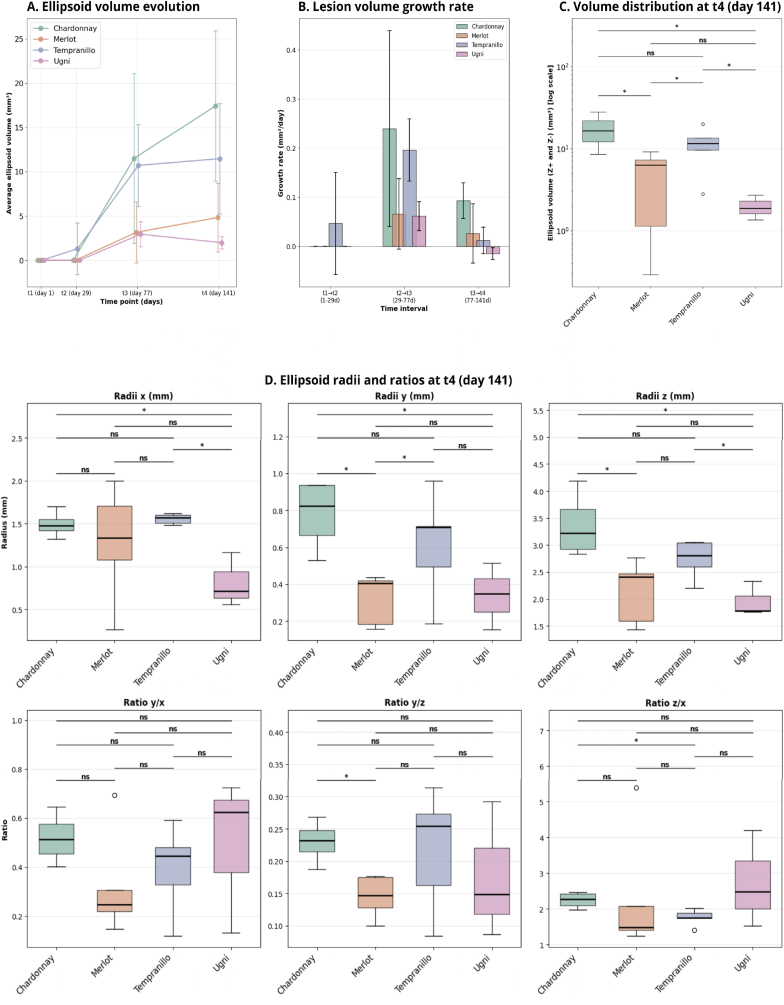
Ellipsoid-based quantification of lesion progression across grapevine varieties under Pch infection. (A) Temporal evolution of average ellipsoid lesion volume for each grapevine variety (Chardonnay, Merlot, Tempranillo, and Ugni) in Pch-infected cuttings according to their relative position on the time axis after inoculation. Points represent group means at each time point, with error bars indicating intra-cultivar variability. (B) Growth rate computation between timepoints (C) Distribution of ellipsoid lesion volumes at day 141 for each variety, displayed on a logarithmic scale. Boxplots show median values, interquartile ranges, and individual outliers. Pairwise statistical comparisons between varieties are indicated above the plots (p < 0.05; ns, not significant). (D) Variety-specific distributions of ellipsoid radii along the three anatomical axes (X, Y, and Z) and their corresponding ratios at Day 141, characterizing lesion anisotropy. Boxplots summarize the distribution of each geometric parameter across specimens, with significance annotations indicating pairwise comparisons.

Differences in lesion development progression among cultivars over time were assessed using statistical analyses of ellipsoid-based lesion volumes at each observation time point, with the temporal spacing of observations in Fig. 8 (A) reflecting the actual intervals after inoculation. With the exception of Tempranillo, no detectable lesion was observed at day 1 and day 29 in the region of interest, indicating that magnetic resonance imaging during the first 30 days post-inoculation provides no meaningful information on lesion formation. This early absence of signal was consistent across cultivars, and notable lesion development only became visible from day 77 onwards.

The development graph across time exhibits two groups: Chardonnay and Tempranillo, versus Merlot and Ugni, the first group showing big lesions and an ongoing development, while the second group shows smaller lesions, and eventually a stalling lesion development.

The lesion growth rate plot shows a slow emergence of lesion progression, with minimal changes observed between the first two timesteps. After the second timestep, the main progression occurs, with Tempranillo and Chardonnay exhibiting higher growth rates (Chardonnay showing the highest variability), and Ugni Blanc and Merlot displaying lower growth rates (Merlot showing the highest variability). After the third timestep, lesion progression was maintained for Chardonnay specimens, whereas it stalled in the other varieties, except for some variability observed within the Merlot specimens. This trend was consistent with the high variability observed for the Merlot variety in the absolute lesion volumes at the final timepoint.

Fig. 8 (C) reveal significative differences between cultivars when comparing the development of lesion at the last time-point. The large and rapidly expanding lesions in Chardonnay and Tempranillo indicate high early susceptibility and weak compartmentalization capacity. Merlot demonstrates a more effective limitation of internal damage, while the lesion spread is highly variable across the specimens, like previously seen in the atlases. Ugni Blanc's minimal lesion volumes suggest enhanced early resistance or more efficient tissue compartmentalization, allowing it to restrict fungal progression even at later infection stages. The coherence between ellipsoid and bounding-box trends confirms that these differences reflect biological variation rather than geometric artifacts.

To test the hypothesis that early lesion development reflects cultivar-specific susceptibility differences, we first formulated the null hypothesis that all varieties exhibit the same lesion volume distribution at the early infection stage. A Kruskal-Wallis test performed on ellipsoid-estimated volumes at Day 141 rejected this null hypothesis (*p* = 0.04) indicating at least one variety differs significantly from the others. This statistically validates the alternate hypothesis that cultivars do not respond uniformly but instead show a distinct level of susceptibility to Pch even at the early stages. The pairwise Kruskal tests (Fig. 8 (C)) show that the cultivar pairs have a significant difference (indicated by their star annotations). Even with the small number of specimens (N = 6), significant differences are observed for all comparisons except for Chardonnay vs Tempranillo and Merlot vs Ugni Blanc, where no statistical differences were observed. Overall, the pattern confirms the presence of distinct susceptibility among cultivars during early infection.

In Fig. 8 (D), the three radius boxplots (x, y, z) in the upper row show clearer cultivar-dependent differences in lesion size than the ratio plots show for lesion shape. Across axes, Chardonnay and Tempranillo generally exhibit the largest median radii (with Chardonnay often highest). Merlot tends to be intermediate but noticeably variable, and Ugni Blanc consistently shows the smallest median radii. Chardonnay and Ugni Blanc consistently show pairwise significant differences in their lesion radii, indicating the strongest separation among the cultivar pairs at t4 (day 141). This suggests that these two cultivars exhibit the most divergent lesion expansion patterns under Pch inoculation compared with the other cultivar combinations. The significant results are concentrated in the absolute radii (x, y, z) rather than in the shape ratios, which suggests that cultivars mainly differ in overall lesion extent rather than in a consistent difference in lesion shape. Significant pairwise differences are seen between Tempranillo and Ugni Blanc in x axis, between Chardonnay - Merlot and Merlot - Tempranillo in y axis, and between Chardonnay - Merlot and Tempranillo - Ugni in z axis. However, when high variability occurs within a cultivar (especially for Merlot), differences cannot be assumed with such a small sample size (N = 6). The fact that most ratio tests are non-significant also fits with lesions mainly differing in overall size across all axes.

It is worth noting that some specimens did not develop any visible lesion during the imaging period and appeared similar to Control plants. No additional information was available to determine whether these vines successfully suppressed the pathogen between Day 1 and Day 29, or whether the inoculation procedure failed for these individuals. These non-responsive specimens were excluded from the statistical analysis to avoid confounding effects, although doing so reduced the sample size and made significance harder to detect. The excluded cases included one Chardonnay, two Merlot, one Tempranillo, and three Ugni Blanc specimens.

Consistent with the volumetric analysis, which show a significant separation of the varieties into two groups with larger (Chardonnay and Tempranillo) versus smaller lesion volumes (Merlot and Ugni Blanc), ellipsoid-derived geometric descriptors (the estimated ellipsoid radii) revealed that this is also reflected along individual dimensions. Chardonnay and Tempranillo exhibited larger extents along r_x_ and r_z_ (Fig. 8 (D) top-left and right) whereas Ugni was significantly smaller along those directions. Differences were also observed in radial lesion depth (r_ᵧ_), with Chardonnay and Tempranillo generally showing deeper penetration compared with Merlot and Ugni Blanc. Merlot showed highly dispersed distributions across all axes, preventing robust statistical discrimination despite occasional large lesions.

When shifting from absolute dimensions to geometric ratios, the grouping became less pronounced. The ratio between r_y_ and r_x_ (Fig. 8 (D) bottom-left) resulted in overlapping distributions across varieties indicating that the radial and depth progression are not discriminant. r_z_/r_x_ (Fig. 8 (D) bottom-right) shows the limitations in the lesion to go through the wood rays relative to its capability to go through the stem axis, without showing any significant discriminant between varieties. Merlot showed an interesting pattern, particularly in the r_y_/r_z_ ratio (Fig. 8 (D), bottom-centre), which was approximately two times lower than that of the other cultivars, indicating a lower mean penetration towards the pith relative to the extent along the cambium axis.

## 4 Discussion

This study demonstrates the potential of high-resolution 3D + t MRI to monitor the earliest stages of fungal colonization in grapevine cuttings, providing functional, non-destructive insight into the tissue functionality during pathogen invasion. Unlike traditional assessments based on external symptoms or destructive sampling, MRI enables continuous observation of internal tissue responses, capturing the onset and progression of lesion formation within days to weeks after inoculation. This early detection capacity is valuable because it reveals anatomical and physiological changes that precede macroscopic symptoms and may be instrumental for understanding host-pathogen interactions.

A key contribution of this work is the geometric processing framework developed to compare lesions across individuals and timepoints. The combination of landmark alignment (standardizes global pose of the specimens), cylindrical unwrapping (stabilizes anatomical correspondences), population atlases (summarizes populations) and double-symmetry geometric ellipsoid modelling (extracts geometrical descriptors) provides a reliable method for normalising inter-individual variability in stem orientation, anatomy, and lesion shape. Although this framework was developed for *Phaeomoniella chlamydospora* infection in grapevine cuttings, it is generalisable to other host-pathogen systems where internal lesion dynamics needs to be quantified across specimens, especially useful (but not limited to) in combination with magnetic resonance imaging if the pathogen progression induces a loss of host tissue function and a change in water status. The ability to align volumes, extract comparable geometric descriptors, and model lesion morphology systematically opens new possibilities for broader comparative pathology studies.

Despite these methodological strengths, several limitations must be acknowledged. While the proof of concept is successful, the sample size remains relatively small, which restricts statistical power, particularly for detecting subtle cultivar differences at early timepoints. The time interval may be considered excessive, especially between t2 (day 29) and t3 (day 77), which diminishes the advantages of 3D + t imaging. During this time interval, most of the lesion progression occurred, suggesting that an additional intermediate timepoint (day 55) would be beneficial, and would probably provide additional discriminative information to distinguish the relative behaviour of the different varieties. Complementary application study on bigger populations with more time points would be interesting to reach a better level of significance. Additionally, the pipeline relies on an assumption of approximate cylindrical symmetry along the longitudinal axis. In mature, field-grown vines, trunk geometry is often non-axisymmetric and branched; therefore, the symmetry-based steps (ellipsoid fit/cylindrical mapping) would not be appropriate and would need to be replaced, along with more advanced registration strategies, including non-linear registration. Nevertheless, longitudinal 3D comparison and lesion quantification remain applicable in principle. Although generally valid for young cuttings, this symmetry is not perfect and may introduce minor biases in the ellipsoid fitting process. The cylindrical transformation worked as intended, though its impact was limited by the localized spread of the fungus in this study, and may prove more helpful with longer monitoring experiments or with other pathogens having a wider transversal development. Furthermore, the functioning tissue MRI signal is inherently variable, with varying intensity levels and patterns due to inter-variety or inter-specimen variation of the water content. This has an influence on the lesion/tissue boundaries automatically segmented by the machine learning model, and ultimately impacts volumetric measurements. These factors could be considered when interpreting small differences between individuals or cultivars.

Even with these limitations, the early MRI-derived lesion metrics revealed clear differences among cultivars. In the spatio-temporal patterns, probabilistic atlas highlighted different development patterns, while showing a good reproducibility level of lesion geometry in Chardonnay, an intermediary trend in Tempranillo and big variations in Merlot and Ugni Blanc. The patterns observed suggest a better early compartmentalization by wood rays in two varieties, while the two others varieties show a capability to limit the inwards progression of the pathogen.

In the study of lesion volume over time, two groups emerged consistently: Chardonnay and Tempranillo showed rapid lesion expansion soon after inoculation, while Merlot and Ugni Blanc displayed slower progression. This separation suggests that early structural degradation captured by MRI reflects meaningful differences in host response. However, this ranking does not fully align with the susceptibility classifications reported in the viticulture literature, where Ugni Blanc is generally described as highly sensitive and Merlot as moderately tolerant. The fact that Tempranillo and Ugni did not follow the expected susceptibility hierarchy is particularly intriguing. These differences are useful because they suggest that early MRI captures cultivar-specific changes that happen before visible chronic symptoms. This creates a practical window for early intervention and treatment testing, and it also provides a complementary tool for phenotyping and screening. These observations open important questions about the sequence of processes that connect initial colonization, host compartmentalization responses, and the later development of chronic trunk disease symptoms. From the perspective of early indicators, our results show that early lesion formation appears useful for comparing how cultivars respond soon after infection in this controlled cutting model. We did observe a statistically significant cultivar effect, but because the sample size is small and variability is high, we consider any cultivar ranking preliminary and in need of confirmation in larger studies. Continuous monitoring over months or seasons would help determine how early physiological signatures translate into later structural degradation and whether the initial MRI signal loss predicts eventual disease outcomes.

Going forward, several developments could strengthen the approach. Higher temporal resolution (smaller timestep) imaging could capture the very early transition between functional tissue and altered one, with a detectable signal loss. Multi-zone analysis separating conductive tissues (xylem vessels) from structural wood (wood rays) may provide a more nuanced understanding of how the fungus perturbs hydraulic function. Finally, integrating geometric lesion metrics with mechanistic modelling of pathogen growth and host responses could help explain how different cultivars regulate or fail to regulate infection spread.

Our work can have practical implications for grapevine health management such as early-stage assessment of internal tissue degradation, where rapid identification of sensitive genotypes can help with planting decisions and guide disease prevention strategies. This framework is not restricted to the four cultivars nor the single pathogen studied here, but can be extended to other perennial woody hosts and to distinct disease mechanisms ranging from wood decaying pathogens to vascular dysfunction. In its current status, our setup is tissue-targeted (35 μm) and therefore best suited to small specimens (1 cm radius). However, it is possible to scale to larger organs or whole plants by using other imaging systems and adapting to mounting strategies to meet both experimental constraints and plant physiological requirements. MRI is particularly strong for distinguishing functional, living, water-rich tissue from water-depleted and non-functional tissue, especially in young tissues of plants. Over longer time scales, plant morphology will change due to growth and hydration dynamics, and this must be taken into account when tracking tissues across timepoints, potentially requiring more advanced longitudinal registration strategies. In addition, while we relied primarily on GE3D in this study, incorporating T1-and T2-weighted contrasts could provide more specific insight into tissue health through disease-related changes in relaxation behavior. Also, while xylem-related features can be visualized in MRI, X-ray CT may be more appropriate when the target is to assess vessel filling or embolism.

In the current phytosanitary context, where the single effective treatment product (arsenite) is restricted in the EU, growers have limited options beyond physically removing infected wood. This work highlights the needs for tools that can detect internal infections before they require destructive interventions, positioning MRI as a non-invasive and dynamic approach. In addition, the quantitative nature of the pipeline makes it a promising benchmark for evaluating the efficacy of new phytosanitary treatments being deployed.

Overall, this work demonstrates that MRI 3D + t, combined with the geometric lesion modelling, offers a powerful framework for non-destructive early-stage monitoring of grapevine trunk disease. It highlights cultivar-dependent early responses and raises questions about how early MRI responses relate to later wood damage. While longer monitoring and larger sample sizes will be needed to connect early patterns to long-term vineyard outcomes, the main added value of the approach is that it makes early internal changes measurable and comparable. This creates a clear path toward practical use as a research tool, both for phenotypic screening for early response traits and to test whether preventive strategies (for example, biocontrol agents or induced-resistance products) can modify early lesion development before severe necrosis becomes established.

## Author contributions

GP: Conceptualization, Methodology, Software, Validation, Formal analysis, Investigation, Data curation, Visualization, Writing - original draft, Writing - review & editing.

MC: Investigation, Resources, Data curation, Methodology, Writing - review & editing.

CGB: Investigation, Resources, Data curation, Methodology, Writing - review & editing.

LLC: Conceptualization, Supervision, Project administration, Funding acquisition, Writing - review & editing.

JLV: Conceptualization, Resources, Supervision, Project administration, Funding acquisition, Writing - review & editing.

CM: Conceptualization, Methodology, Investigation, Resources, Supervision, Project administration, Funding acquisition, Writing - review & editing.

RF: Conceptualization, Methodology, Software, Validation, Formal analysis, Visualization, Supervision, Funding acquisition, Writing - original draft, Writing - review & editing.

## Funding

This work was supported by the French Ministry of Agriculture and Food, France AgriMer, the Comité National des Interprofessions des Vins à appellation d'origine et à indication géographique (CNIV), and the Institut Français de la Vigne et du Vin (IFV) within VITIMAGE-2024 and SMIYC projects (program Plan National Dépérissement du Vignoble); and by Agropolis fondation-APLIM Etendard project (contract 1504-005). Imaging acquisitions were performed at the BioNanoNMRI platform member of the national infrastructure France-BioImaging supported by the French National Research Agency « Investments for the Future» (ANR-10-INBS-04), and of the Labex CEMEB (ANR-10-LABX-0004) and NUMEV (ANR-10-LABX-0020).

## Declaration of competing interest

The authors declare that there is no conflict of interest regarding the publication of this article.

## Data Availability

The datasets generated and analyzed in this study include approximately 160 GB of raw 3D magnetic resonance images and 1.4 TB of processed data products. Due to the large volume of these files and current storage and transfer constraints, the data are available from the corresponding author upon reasonable request. The processing pipeline (including scripts and parameters required to reproduce the processed outputs from the raw data) is available at https://doi.org/10.5281/zenodo.17944369.

## References

[1] Beris E., Selim M., Kechagia D., Evangelou A. (2023).

[2] Kenfaoui J., Radouane N., Mennani M., Tahiri A., El Ghadraoui L., Belabess Z., Fontaine F., El Hamss H., Amiri S., Lahlali R. (2022). A panoramic view on grapevine trunk diseases threats: case of Eutypa dieback, Botryosphaeria dieback, and Esca disease. J. Fungi.

[3] Bendel N., Kicherer A., Backhaus A., Klück H.C., Seiffert U., Fischer M., Voegele R.T., Töpfer R. (2020). Evaluating the suitability of hyper- and multispectral imaging to detect foliar symptoms of the grapevine trunk disease Esca in vineyards. Plant Methods.

[4] Levasseur-Garcia C., Malaurie H., Mailhac N. (2016). An infrared diagnostic system to detect causal agents of grapevine trunk diseases. J. Microbiol. Methods.

[5] Vaz A.T., Del Frari G., Chagas R., Ferreira A., Oliveira H., Boavida Ferreira R. (2020). Precise non-destructive location of defective woody tissue in grapevines affected by wood diseases. Phytopathol. Mediterr..

[6] Milien M., Renault-Spilmont A.S., Cookson S.J., Sarrazin A., Verdeil J.L. (2012). Visualization of the 3D structure of the graft union of grapevine using X-ray tomography. Sci. Hortic..

[7] Fernandez R., Le Cunff L., Mérigeaud S., Verdeil J.L., Perry J., Larignon P., Spilmont A.S., Chatelet P., Cardoso M., Goze-Bac C. (2024). End-to-end multimodal 3D imaging and machine learning workflow for non-destructive phenotyping of grapevine trunk internal structure. Sci. Rep..

[8] Borisjuk L., Neuberger T. (2025). The look insight–magnet resonance imaging (MRI) of the inner life of plants. J. Plant Physiol..

[9] Boulc’h P.N., Collewet G., Guillon B., Stéphane Q., Laurent L., Maja M. (2024). Quantitative MRI imaging of parenchyma and venation networks in Brassica napus leaves: effects of development and dehydration. Plant Methods.

[10] Blystone S., Nuixe M., Traoré A.S., Cochard H., Picon-Cochard C., Pagés G. (2024). Towards portable MRI in the plant sciences. Plant Methods.

[11] Buy S., Le Floch S., Tang N., Sidiboulenouar R., Zanca M., Canadas P., Nativel E., Cardoso M., Alibert E., Dupont G., Ambard D. (2018). Flip-flop method: a new T1-weighted flow-MRI for plants studies. PLoS One.

[12] Borisjuk L., Neuberger T., Rolletschek H. (2025). Lipid MRI in plant science: principles and potential areas of application. J. Exp. Bot..

[13] Meixner M., Tomasella M., Foerst P., Windt C.W. (2020). A small-scale MRI scanner and complementary imaging method to visualize and quantify xylem embolism formation. New Phytol..

[14] Van Dusschoten D., Metzner R., Kochs J., Postma J.A., Pflugfelder D., Bühler J., Schurr U., Jahnke S. (2016). Quantitative 3D analysis of plant roots growing in soil using magnetic resonance imaging. Plant Physiol..

[15] Pflugfelder D., Metzner R., Van Dusschoten D., Reichel R., Jahnke S., Koller R. (2017). Non-invasive imaging of plant roots in different soils using magnetic resonance imaging (MRI). Plant Methods.

[16] Van As H., Scheenen T., Vergeldt F.J. (2009 Dec). MRI of intact plants. Photosynth. Res..

[17] Selim D.A., Nassar R.M., Boghdady M.S., Bonfill M. (2019). Physiological and anatomical studies of two wheat cultivars irrigated with magnetic water under drought stress conditions. Plant Physiol. Biochem..

[18] Collins D.L., Zijdenbos A.P., Kollokian V., Sled J.G., Kabani N.J., Holmes C.J., Evans A.C. (2002). Design and construction of a realistic digital brain phantom. IEEE Trans. Med. Imag..

[19] Calixto C., Dorigatti Soldatelli M., Jaimes C., Pierotich L., Warfield S.K., Gholipour A., Karimi D. (2025). A detailed spatiotemporal atlas of the white matter tracts for the fetal brain. Proc. Natl. Acad. Sci..

[20] Noorizadeh N., Kazemi K., Taji S.M., Danyali H., Aarabi A. (2024). Subject-specific atlas for automatic brain tissue segmentation of neonatal magnetic resonance images. Sci. Rep..

[21] Wolz R., Chu C., Misawa K., Fujiwara M., Mori K., Rueckert D. (2013). Automated abdominal multi-organ segmentation with subject-specific atlas generation. IEEE Trans. Med. Imag..

[22] Neumann M., Xu X., Smaczniak C., Schumacher J., Yan W., Blüthgen N., Greb T., Jönsson H., Traas J., Kaufmann K. (2022). A 3D gene expression atlas of the floral meristem based on spatial reconstruction of single nucleus RNA sequencing data. Nat. Commun..

[23] Marchi G. (2001). Susceptibility to Esca of various grapevine ("Vitis vinifera") cultivars grafted on different rootstock in a vineyard in the Province of Siena (Italy). Phytopathol. Mediterr..

[24] Fischer M., Ashnaei S.P. (2019). Grapevine, esca complex, and environment: the disease triangle. Phytopathol. Mediterr..

[25] Martín L., Fontaine F., Castaño F.J., Songy A., Roda R., Vallet J., Ferrer-Gallego R. (2019). Specific profile of Tempranillo grapevines related to Esca-leaf symptoms and climate conditions. Plant Physiol. Biochem..

[26] Bruez E., Lecomte P., Grosman J., Doublet B., Bertsch C., Fontaine F., Ugaglia A., Teissedre P.L., Da Costa J.P., Guerin-Dubrana L. (2013). Overview of Grapevine Trunk Diseases in France in the 2000s. Phytopathol. Mediterr..

[27] Lecomte P., Darrieutort G., Liminana J.M., Comont G., Muruamendiaraz A., Legorburu F.J., Choueiri E., Jreijiri F., El Amil R., Fermaud M. (2012). New insights into esca of grapevine: the development of foliar symptoms and their association with xylem discoloration. Plant Dis..

[28] Pouzoulet J., Scudiero E., Schiavon M., Rolshausen P.E. (2017). Xylem vessel diameter affects the compartmentalization of the vascular pathogen Phaeomoniella chlamydospora in grapevine. Front. Plant Sci..

[29] Péros J.P., Berger G. (1994). A rapid method to assess the aggressiveness of Eutypa lata isolates and the susceptibility of Grapevine cultivars to Eutypa dieback. Agronomie.

[30] Moisy C., Berger G., Flutre T., Le Cunff L., Péros J.P. (2017). Quantitative assessment of grapevine wood colonization by the dieback fungus Eutypa lata. J. Fungi.

[31] Phukon G., Fernandez R. (2025).

[32] Fernandez R., Moisy C. (2021). Fijiyama: a registration tool for 3D multimodal time-lapse imaging. Bioinformatics.

[33] Srinivasan P.P., Kim L.A., Mettu P.S., Cousins S.W., Comer G.M., Izatt J.A., Farsiu S. (2014). Fully automated detection of diabetic macular edema and dry age-related macular degeneration from optical coherence tomography images. Biomed. Opt. Express.

[34] Paulson B., Lee S., Kim Y., Moon Y., Kim J.K. (2019). Miniaturized omnidirectional flexible side-view endoscope for rapid monitoring of thin tubular biostructures. Biomed. Opt. Express.

[35] Cui Z., Feng J., Zhou J. (2023). Monocular 3D fingerprint reconstruction and unwarping. IEEE Trans. Pattern Anal. Mach. Intell..

[36] Arganda-Carreras I., Kaynig V., Rueden C., Eliceiri K.W., Schindelin J., Cardona A., Sebastian Seung H. (2017). Trainable Weka Segmentation: a machine learning tool for microscopy pixel classification. Bioinformatics.

