# Supplementary material for: Atlas-based spatiotemporal MRI phenotyping of 3D fungal spread in grapevine wood
Source: Plant Phenomics. 2026 Mar 30;8(2):100185. doi: 10.1016/j.plaphe.2026.100185 (PMC13316239; doi:10.1016/j.plaphe.2026.100185)
Supplement: Multimedia component 1 [file mmc1.docx]

## **SUPPLEMENTARY MATERIALS**

Note S1


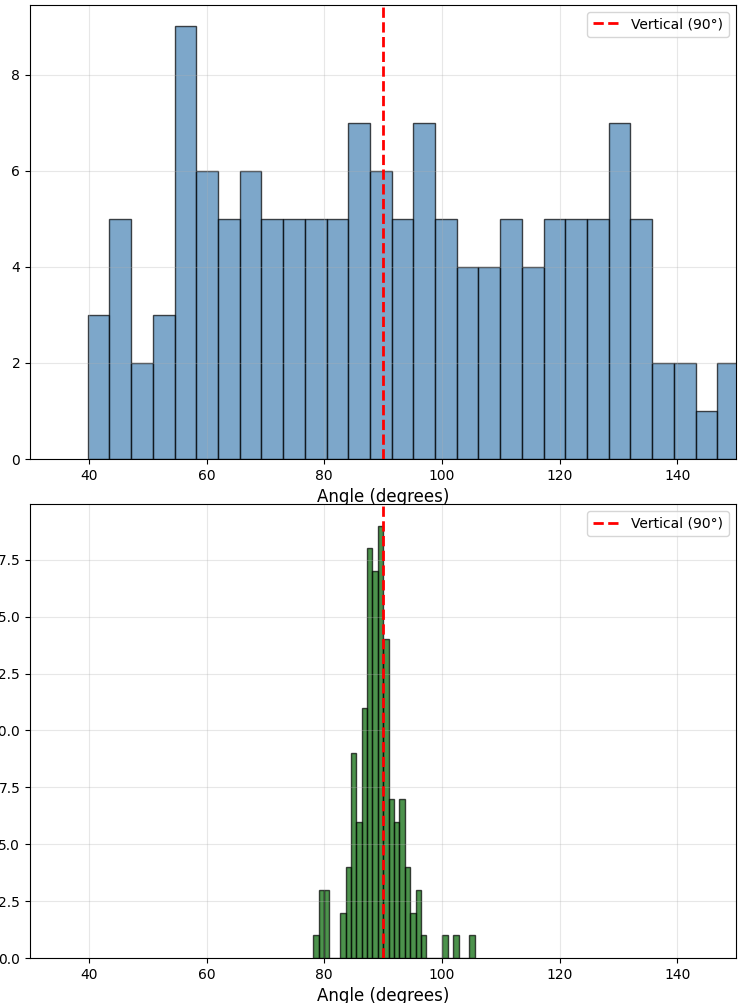

**Fig. S1**: Wood rays angle distribution - before (blue) and after (green) transformation


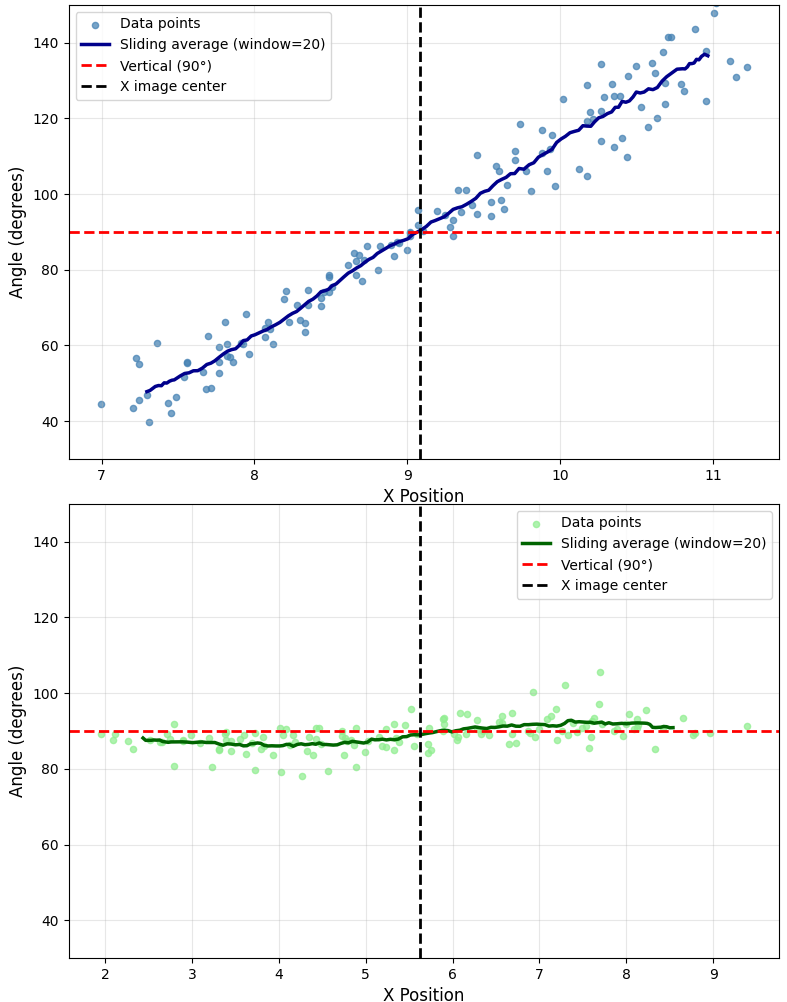


**Fig. S2**[: Angle vs X Position – before (blue) and after (green) transformation](https://www.zotero.org/google-docs/?xi0VIe)
